# *SETD8^C302R^* Mutation Revealed from Myofibroblastoma‐Discordant Monozygotic Twins Leads to p53/p21 Deficit and WEE1 Inhibitor Sensitivity

**DOI:** 10.1002/advs.202001041

**Published:** 2020-08-05

**Authors:** Miao Li, Hongwu Wang, Hongwei Liao, Jiaxin Shen, Yinfang Wu, Yanping Wu, Qingyu Weng, Chen Zhu, Xinwei Geng, Fen Lan, Yang Xia, Bin Zhang, Hang Zou, Nan Zhang, Yunzhi Zhou, Zhihua Chen, Huahao Shen, Songmin Ying, Wen Li

**Affiliations:** ^1^ Key Laboratory of Respiratory Disease of Zhejiang Province, Department of Respiratory and Critical Care Medicine Second Affiliated Hospital of Zhejiang University School of Medicine Hangzhou Zhejiang 310009 China; ^2^ Department of Respiratory and Critical Care Medicine Emergency General Hospital Beijing 100028 China

**Keywords:** genome instability | p53/p21 | rare mutations | SETD8 | WEE1 inhibition

## Abstract

High‐throughput gene sequencing has identified various genetic variants as the culprits for some common hereditary cancers. However, the heritability of a substantial proportion of cancers remains unexplained, which may result from rare deleterious mutations hidden in a myriad of nonsense genetic variations. This poses a great challenge to the understanding of the pathology and thus the rational design of effective treatments for affected patients. Here, whole genome sequencing is employed in a representative case in which one monozygotic twin is discordant for lung inflammatory myofibroblastoma to disclose rare tumor‐related mutations. A missense single nucleotide variation rs61955126 T>C in the lysine methyltransferase SETD8 (accession: NM_020382, *SETD8^C302R^*) is exposed. It is shown that SETD8 is vital for genomic integrity by promoting faithful DNA replication, and its C302R mutation downregulates the p53/p21 pathway. Importantly, the *SETD8^C302R^* mutation significantly increases the sensitivity of cancer cells to WEE1 inhibition. Given that WEE1 inhibitors have shown great promise for clinical approval, these results impart a potential therapeutic approach using WEE1 inhibitor for cancer patients carrying the same mutation, and indicate that genome sequencing and genetic functional studies can be integrated into individualized therapies.

## Graphical abstract

Whole genome sequencing on a pair of identical twins discordant for inflammatory myofibroblastoma and following functional studies reveal a missense single nucleotide variation (rs61955126 T>C) in the lysine methyltransferase SETD8 (accession: NM_020382, *SETD8^C302R^*). SETD8 promotes faithful DNA replication and genomic integrity, whereas *SETD8^C302R^* mutation downregulates the p53/p21 pathway and increases sensitivity of cancer cells to WEE1 inhibition.

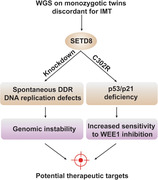

## 1 Introduction

Cancer is in essence a disease resulting from the interaction between genetic and environmental factors. This process is driven by numerous genetic variations arising from spontaneous mutations or harmful environmental exposure, ranging from single nucleotide mutations to whole‐chromosomal changes.^[^ ^1^, ^2^, ^3^, ^4^ ^]^ Underlying these genetic alterations is the genomic instability caused by the breakdown in one or several components of the genomic maintenance machinery,^[^ ^5^ ^]^ which creates the propensity to acquisition and accumulation of genomic mutation and promotes tumor progression.^[^ ^6^ ^]^ Paradoxically, these mutations also provide targets for therapy.^[^ ^7^, ^8^ ^]^

Decades of researches have revealed an array of mutations that are considered to be culprits for cancer development throughout large populations, which drive cellular transformation by sustaining growth signals and/or facilitating genetic evolution.^[^ ^4^, ^9^, ^10^, ^11^, ^12^, ^13^, ^14^ ^]^ However, for those patients carrying rare mutations that are less understood, it is difficult to benefit from current therapeutics that focus on more common genetic abnormalities. Thus, individualized treatments are needed that are based on our understanding of tumor‐initiating genetic alterations and their biological consequences. In fact, by virtue of high‐throughput sequencing technologies that can efficiently reveal germline and/or somatic mutations,^[^ ^15^, ^16^ ^]^ individualized therapies are becoming increasingly practical.

Monozygotic (MZ) twins discordant for cancer are especially suitable for such genetic studies because these individuals arise from a single cell, inheriting almost identical genetic material, and share nearly the same growth environments before and after birth. Analysis of MZ twins averts interferences from genetic background, early life‐environment exposure, age, and gender, and provides a unique opportunity to investigate genomic variants that contribute to risks of various diseases including cancer, autoimmune diseases, chronic inflammation, and neurological disorders.^[^ ^17^, ^18^, ^19^, ^20^, ^21^, ^22^ ^]^ This type of investigation can facilitate understanding of disease pathology and unravel potential targets for pharmacological intervention, promoting rational design and development of novel therapies.

Here, in order to identify rare tumor‐related genomic alterations for further functional validation and therapeutic targeting studies, we applied whole genome sequencing (WGS) on a pair of MZ twins discordant for lung inflammatory myofibroblastoma (IMT), a rare mesenchymal neoplasm that accounts for <1% of lung neoplasms.^[^ ^23^, ^24^, ^25^ ^]^ Genetic screening and functional studies of candidate mutations led to a single nucleotide variation of lysine methyltransferase SETD8 (*SETD8^C302R^*) that resulted in dysfunctional p53/p21 pathway and increased sensitivity to WEE1 inhibition. Compared with preexisting literatures, our work integrated genome sequencing and drug sensitivity screening, which for the first time revealed the association between the rare *SETD8^C302R^* and IMT while presenting a potential therapeutic agent for *SETD8^C302R^* cancer cells. Considering that WEE1 inhibitors are currently undergoing phase II clinical trials (NCT02666950, NCT02196168, NCT02101775, NCT01164995, NCT02194829, NCT03668340, NCT03718143, NCT02037230, NCT03253679, NCT04439227, NCT02688907, NCT02593019, NCT03330847, NCT02087241, NCT02095132, and NCT03385655), our findings hold promise to be eventually translated for future patients carrying the same mutation.

## 2 Results

### 2.1 Genetic Sequencing of a Monozygotic Twin Pair Discordant for Myofibroblastoma

**Figure 1:**
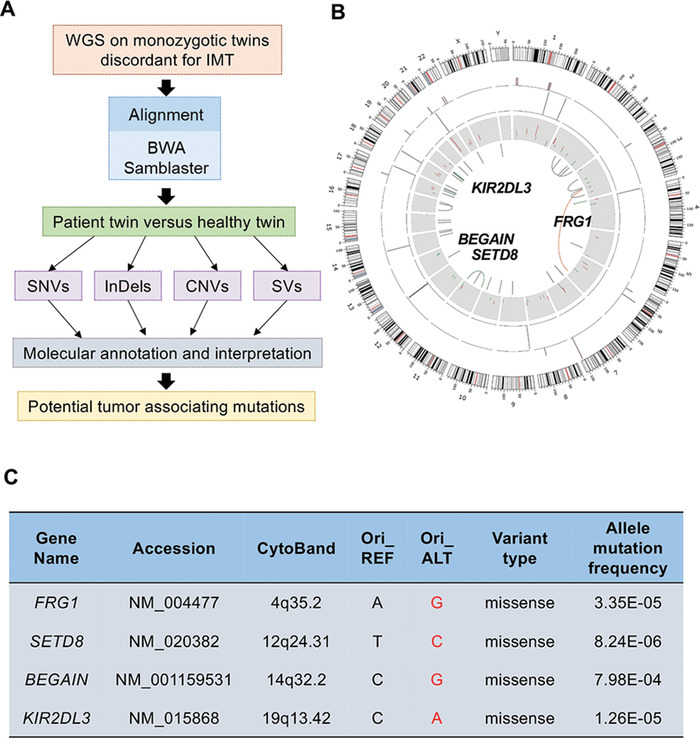
WGS and mutational analysis of a MZ twin pair discordant for myofibroblastoma. A) Process to analyze WGS data from the MZ twin pair discordant for myofibroblastoma. B) Circos plot showing the genetic alterations identified in the patient, with her healthy twin as a germline and normal control. The outermost circle (first circle) is the chromosome information. The second circle indicates the density of SNVs. The third circle indicates the density of InDels. The fourth circle indicates CNVs by Control‐FREEC. The fifth circle indicates SVs by Crest. Four SNVs with general mutation frequency lower than one in 70 000 were marked. C) Genetic events of four SNVs in (B) and annotated by ANNOVAR. Red words represent mutations in the patient.

In order to identify potential pathogenic genomic mutations, WGS was applied to the MZ twin pair discordant for myofibroblastoma. The growth environment and life‐environment exposure of the MZ twins were very similar before they were presented to hospital. Genomic alterations that include single nucleotide variants (SNVs), insert and deletion (InDels), copy number alterations (CNAs), and structural variants (SVs) were detected. With the healthy twin as a control, we preserved the patient's specific mutations (**Figure** **1**A,B). Further, missense SNVs in gene bodies with mutation frequencies lower than one in 70 000 according to 1000 Genomes^[^ ^26^ ^]^ and the Exome Aggregation Consortium database were screened and scored using SIFT, PolyPhen, MutationTaster, and CADD. To perform functional studies on candidate rare tumor drivers (Figure 1C), specific siRNAs targeting the four genes were employed to transfect human osteosarcoma cells (U2OS) and human lung cancer cells (A549) (Figure S1A, Supporting Information), which revealed that only SETD8 knockdown retarded cell proliferation and induced increases in apoptosis (Figure S1B–E, Supporting Information). Therefore, we proceeded to look into the biological consequences of SETD8 deficiency.

### 2.2 SETD8 Is Essential for Maintaining Genome Integrity

**Figure 2:**
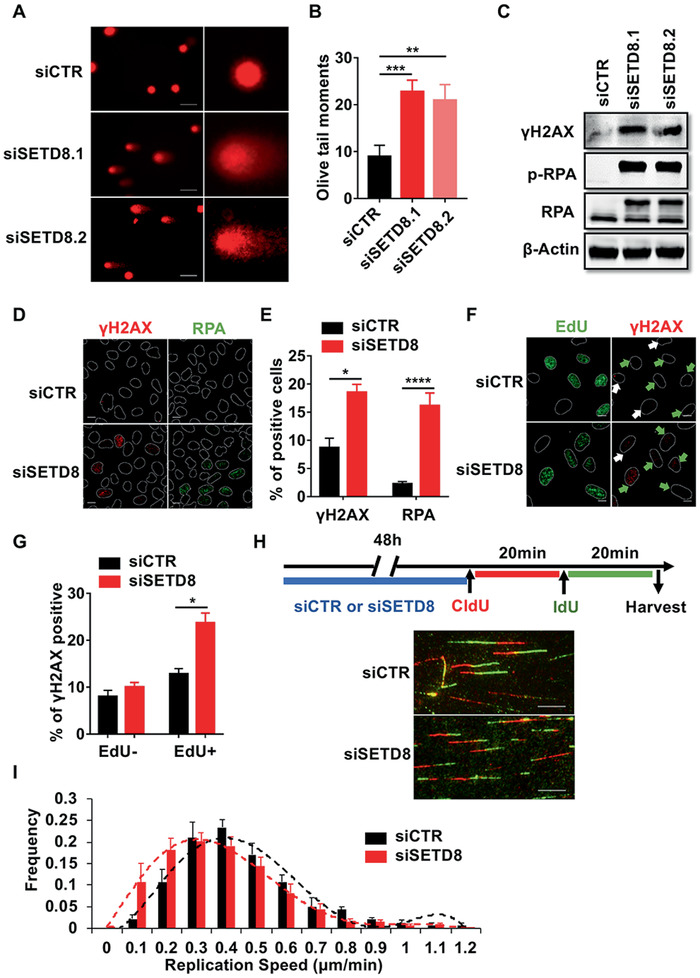
SETD8 is essential for maintaining genome integrity. A) Representative images of comet assays in U2OS cells treated with 10 × 10^−9^ m siSETD8 or control siRNA for 48 h (scale bar, 50 µm). B) Quantification of the data from (A) (at least 60 cells in each group from three independent samples). C) Immunoblot analysis for *γ*H2AX, RPA, and p‐RPA in whole cell extracts from U2OS cells treated as (A). *β*‐Actin was used as a loading control. D) Representative images of *γ*H2AX and RPA foci in U2OS cells treated with siSETD8.2 for 48 h (scale bar, 10 µm). E) Quantification of the data from (D). Cells with over five *γ*H2AX or RPA foci were counted as positive cells (at least three independent samples). F) Representative images of *γ*H2AX in replicating and non‐replicating cells treated as (D). Green arrows represent replicating cells and white arrows represent non‐replicating cells (scale bar, 10 µm). G) Quantification of the data from (F). Replicating cells were pulse‐labeled with 10 × 10^−6^ m EdU for 30 min. Cells with over five *γ*H2AX foci were counted as positive cells (*n* = 3 independent samples). H) Experimental procedure and representative images of ongoing replication forks in U2OS cells treated as (D) (scale bar, 10 µm). I) Quantification of the replication speeds from (H) (1500 fibers in each group from three independent samples). Data are presented as means ± SEM. For (B), (E), and (G), significance was determined by two‐tailed Student's *t*‐tests. *p < 0.05; **p < 0.01; ***p < 0.001; ****p < 0.0001.

Because SETD8 depletion was found to increase cell apoptosis, we first determined whether DNA breaks were elevated after SETD8 knockdown by comet assays (**Figure** **2**A,B; Figure S2A, Supporting Information). As shown in Figure 2A, 48 h after two independent SETD8 siRNAs transfections, SETD8‐depleted cells were found to have longer comet tails, suggesting that spontaneous DNA damage may occur in cells lacking SETD8. To further confirm this observation, we measured the DNA damage response (DDR) proteins using immunoblot and immunofluorescence assays after SETD8 depletion. Results from these studies indicated that the *γ*H2AX and RPA foci formation, as well as RPA hyper‐phosphorylation, was evidently increased in SETD8 knockdown cells (Figure 2C–E). Similar results were found in normal and transformed human bronchial epithelial (HBE) cells (Figure S2B,C, Supporting Information), suggesting that these were not cell type specific. To determine whether the DNA damage caused by SETD8 silencing was replication‐associated, we pulse‐labeled the siSETD8‐transfected cells with EdU to mark replicating cells.^[^ ^27^ ^]^ *γ*H2AX foci predominantly formed in EdU‐positive cells (Figure 2F,G), suggesting a role for SETD8 in the prevention of replication‐born genomic instability. To address this question, we conducted DNA fiber assays by pulse labeling siSETD8‐treated cells sequentially with the thymidine analogs chlorodeoxyuridine (CldU) and iododeoxyuridine (IdU). The replication fork speed was reduced in cells lacking SETD8 (Figure 2H,I). Taken together, these results suggest that SETD8 plays a role in maintaining genome integrity by ensuring faithful DNA replication.

### 2.3 Patient‐Derived *SETD8^C302R^* Mutation Does Not Affect Genome Integrity Directly

**Figure 3:**
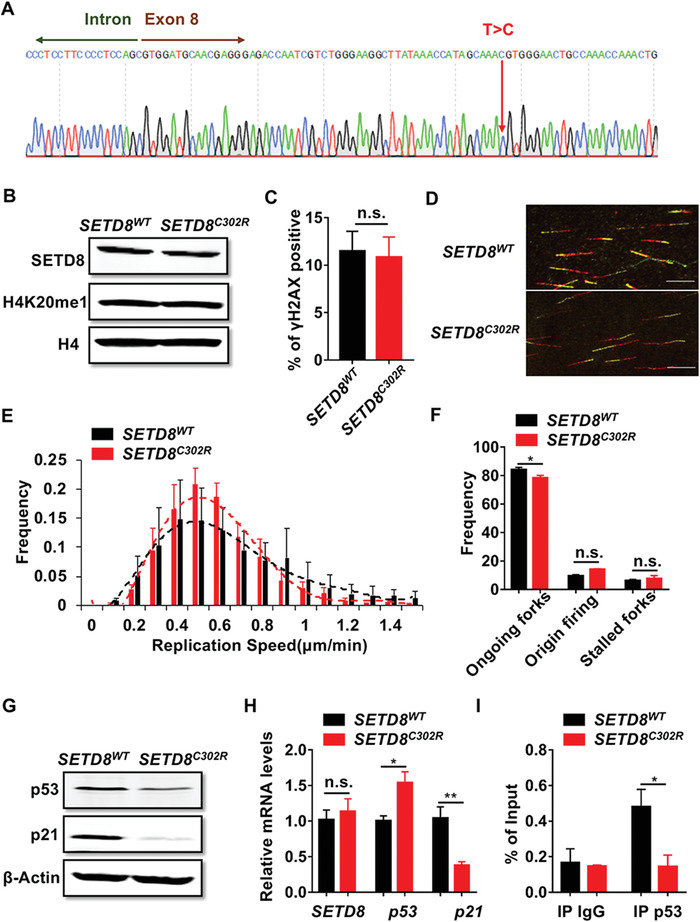
Patient‐derived *SETD8^C302R^* mutation downregulates the p53/p21 pathway. A) Sequencing data of the *SETD8^C302R^* mutant clone built using CRISPR/Cas9; red arrow represents the single nucleotide variation rs61955126 T>C. B) Immunoblot analysis for the indicated proteins in whole cell extracts from *SETD8^WT^* and *SETD8^C302R^* mutant cells. Histone H4 was used as a loading control. C) Immunofluorescence analysis of *γ*H2AX foci in *SETD8^WT^* and *SETD8^C302R^* mutant cells. Cells with over five *γ*H2AX foci were counted as positive cells (*n* = 3 independent samples). D–F) Replication forks analysis of *SETD8^WT^* and *SETD8^C302R^* mutant cells continuously labeled with CldU and IdU for 20 min. D) Representative images of ongoing replication forks in *SETD8^WT^* and *SETD8^C302R^* mutant cells (scale bar, 10 µm). E) Quantification of fork speeds (1500 fibers in each group from three independent samples). F) Quantification of the rates of stalled forks and new origin firing (*n* = 3 independent samples). G) Immunoblot analysis of p53 and p21 in *SETD8^WT^* and *SETD8^C302R^* mutant cells. *β*‐Actin was used as a loading control. H) Relative mRNA levels of *SETD8*, *p53*, and *p21* in *SETD8^WT^* and *SETD8^C302R^* mutant cells quantified by qPCR. *β*‐Actin was used as an internal control (at least three independent samples). I) ChIP analysis of the occupancy of p53 at the *p21* promoter in *SETD8^WT^* and *SETD8^C302R^* mutant cells. Normal mouse IgG was used as a negative control (*n* = 3 independent samples). Data are presented as means ± SEM. Two‐tailed Student's *t*‐tests were performed in (C) and (H), and two‐way ANOVA followed by Sidak's multiple comparison post‐tests were performed in (F) and (I). *p < 0.05; **p < 0.01; n.s.: not significant.

As implicated in our data and previous studies, SETD8 plays a role in maintaining genome integrity.^[^ ^28^, ^29^, ^30^ ^]^ *SETD8^C302R^* derived from the patient is located in the catalytic domain of the enzyme. To better understand the functional consequences, we employed CRISPR/Cas9 technology to construct a cell line harboring the *SETD8^C302R^* point mutation based on U2OS (**Figure** **3**A). We examined the cell proliferation and apoptosis (Figure S3A–D, Supporting Information), as well as the protein levels of SETD8 and the monomethylation of its main substrate histone H4 (H4K20me1), and found no differences between *SETD8^WT^* and *SETD8^C302R^* mutant cells (Figure 3B). Because SETD8 deficiency caused genome instability, we investigated whether the *SETD8^C302R^* mutation also affects genome integrity. DDR marker *γ*H2AX was measured using immunofluorescence, but no evident difference was observed between *SETD8^WT^* and *SETD8^C302R^* mutant cells (Figure 3C). We also detected DNA replication speed by DNA fiber assays. The *SETD8^C302R^* mutant cells appeared to have normal replication forks as did the *SETD8^WT^* cells (Figure 3D–F). Therefore, the *SETD8^C302R^* mutation does not lead to genome instability directly.

### 2.4 *SETD8^C302R^* Mutation Downregulates the p53/p21 Pathway

SETD8 has been shown to monomethylate non‐histone protein substrates such as tumor suppressor p53.^[^ ^31^, ^32^ ^]^ To investigate whether the *SETD8^C302R^* mutation affects p53 and its downstream effectors, we examined the protein levels of p53 and its regulatory target p21 in *SETD8^WT^* and *SETD8^C302R^* cells. *SETD8^C302R^* only slightly decreased p53 expression, but resulted in a marked reduction in p21 level (Figure 3G). The mRNA levels of *p53* and *p21* were then measured. In *SETD8^C302R^* mutant cells, *p53* mRNA level was increased while *p21* mRNA was clearly downregulated (Figure 3H), which raised the possibility that the p21 reduction in *SETD8^C302R^* mutant cells resulted from insufficient p53, even though the increased *p53* mRNA level might to some extent compensate for that deficiency. To prove this, we examined the binding of p53 to the *p21* promoter in *SETD8^WT^* and *SETD8^C302R^* cells by chromatin immunoprecipitation (ChIP) assays, and found there was less p53 binding to the *p21* promoter in *SETD8^C302R^* mutant cells than that in *SETD8^WT^* cells (Figure 3I). All these suggest that the *SETD8^C302R^* mutation can lead to incompetence of the p53/p21 pathway.

### 2.5 *SETD8^C302R^* Mutant Cells Display Increased Sensitivity to WEE1 Inhibition

**Figure 4:**
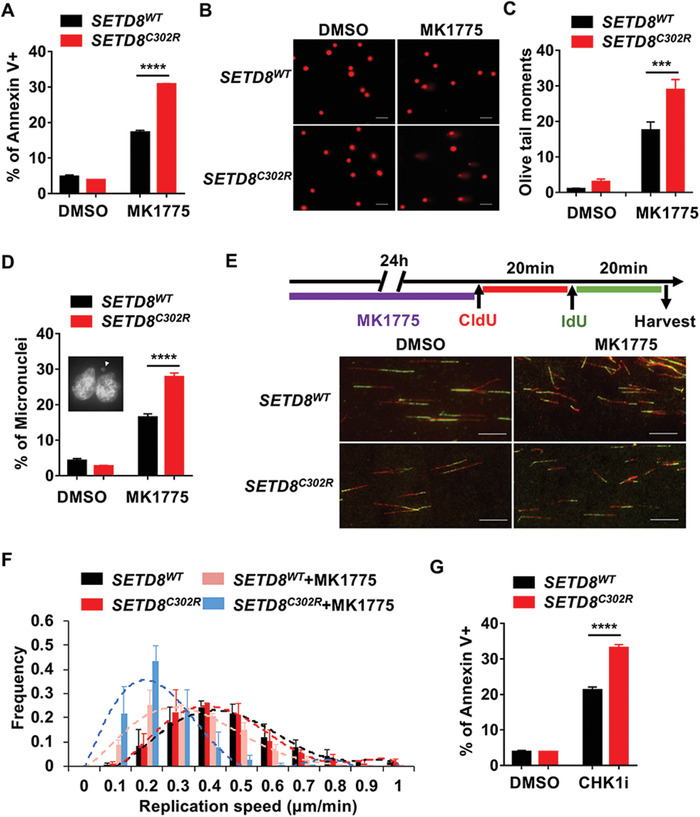
*SETD8^C302R^* mutant cells display increased sensitivity to WEE1 inhibition. A) Apoptosis analysis of *SETD8^WT^* and *SETD8^C302R^* mutant cells treated with 200 × 10^−9^ m MK1775 for 48 h by flow cytometry. Apoptotic cells were labeled with annexin V (*n* = 6 independent samples). B) Representative images of the comet assays in cells treated with 200 × 10^−9^ m MK1775 for 12 h (scale bar, 50 µm). C) Quantification of comet assays from (B). Olive tail moments were measured by CaspLab (at least 160 cells in each group from three independent samples). D) Representative image and quantification of micronuclei in cells treated as (B) (at least three independent samples). E) Experimental procedure and representative images of ongoing replication forks in cells treated with 200 × 10^−9^ m MK1775 for 24 h (scale bar, 10 µm). F) Quantification of the replication speeds from (E) (at least 240 fibers in each group from three independent samples). G) Apoptosis analysis of *SETD8* ^*WT*^and *SETD8* ^*C302R*^mutant cells treated with 2 × 10^−6^ M CHK1 inhibitor for 48 h by flow cytometry. Apoptotic cells were labeled with annexin V (n = 3 independent samples). Data are presented as means ± SEM. Two‐way ANOVA followed by Sidak's multiple comparison post‐tests were performed in (A), (C), (D), and (G). ***p < 0.001; ****p < 0.0001.

Because p21 was reported to play an essential role in fork progression,^[^ ^33^, ^34^ ^]^ we tested whether *SETD8^C302R^* cells are vulnerable to genotoxic agents. *SETD8^C302R^* cells were treated with cisplatin, camptothecin, and WEE1 inhibitor MK1775 (Figure S4, Supporting Information; **Figure** **4**A). Interestingly, *SETD8^C302R^* cells were the most sensitive to MK1775, which is a pyrazole‐pyrimidine derivative and ATP‐competitive small molecule that targets the kinase activity of WEE1.^[^ ^35^ ^]^ Consistently, we found that upon exposure to MK1775, *SETD8^C302R^* mutant cells exhibited increased micronuclei formation and DNA damage (Figure 4B–D). Moreover, in the presence of MK1775, the DNA replication speed of *SETD8^C302R^* mutant cells was found to be slower than that of *SETD8^WT^* cells (Figure 4E,F). These data suggest that *SETD8^C302R^* mutant cells are more sensitive to WEE1 inhibitor than *SETD8^WT^* cells due to exacerbated genome instability related to DNA replication. In support of this hypothesis, *SETD8^C302R^* cells were found to have similar sensitivity to inhibition of CHK1, which is also an essential genome caretaker (Figure 4G).

### 2.6 The Increased Sensitivity of *SETD8^C302R^* Mutant Cells to WEE1 Inhibition Can Be Rescued by p53/p21 Stabilization

**Figure 5:**
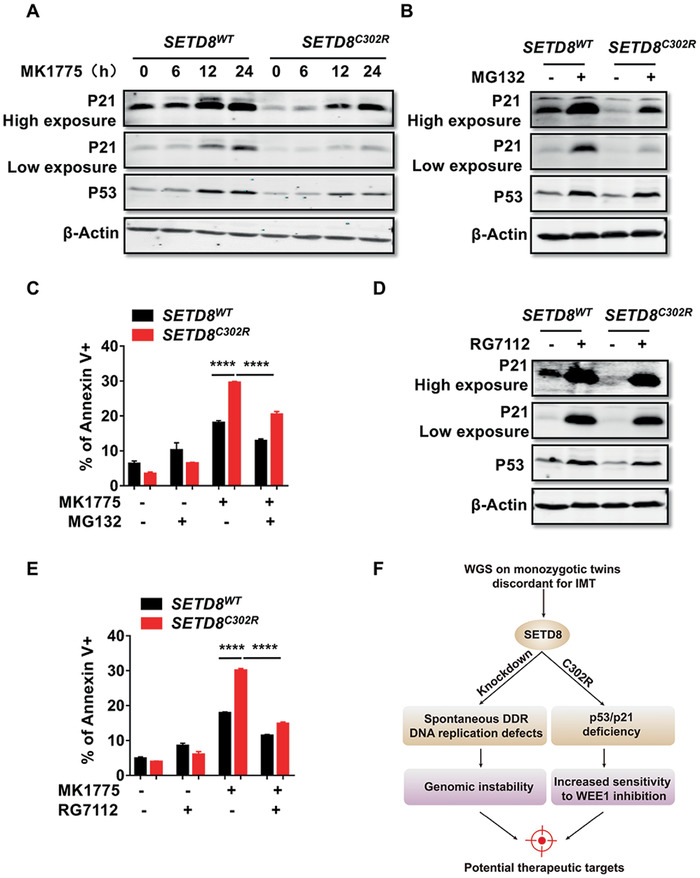
The increased sensitivity of *SETD8^C302R^* mutant cells to WEE1 inhibition can be rescued by p53/p21 stabilization. A) Immunoblot analysis of p53 and p21 in *SETD8^WT^* and *SETD8^C302R^* mutant cells treated with 200 × 10^−9^ m MK1775. *β*‐Actin was used as a loading control. B) Immunoblot analysis of p53 and p21 in *SETD8^WT^* and *SETD8^C302R^* mutant cells treated with 5 × 10^−6^ m MG132 for 4 h. C) Apoptosis analysis of cells treated with 200 × 10^−9^ m MK1775 after MG132 treatment for another 48 h by flow cytometry (*n* = 6 independent samples). D) Immunoblot analysis of p53 and p21 in *SETD8^WT^* and *SETD8^C302R^* mutant cells treated with 2 × 10^−6^ m RG7112 for 48 h. E) Apoptosis analysis of cells treated with 200 × 10^−9^ m MK1775 and 2 × 10^−6^ m RG7112 for 48 h by flow cytometry (*n* = 6 independent samples). F) A schematic representation of our studies regarding SETD8. The absence of SETD8 leads to genome instability, and *SETD8^C302R^* mutation from the patient downregulates the p53/p21 pathway and sensitizes tumor cells to WEE1 inhibition. Data are presented as means ± SEM. Two‐way ANOVA followed by Tukey's multiple comparison post‐tests were performed in (C) and (E). ****p < 0.0001.

p53 and p21 are master regulators in response to various types of cellular damage. Thus, we examined whether p53 and p21 expression could be effectively induced by MK1775 treatment in *SETD8^C302R^* mutant cells. Upon addition of MK1775, the protein levels of p53 and p21 in *SETD8^C302R^* cells were elevated, but to a much less extent than that measured in *SETD8^WT^* cells (**Figure** **5**A). Next, we investigated whether stabilizing p53 in *SETD8^C302R^* mutant cells would mitigate sensitivity to MK1775. The proteasome inhibitor MG132 and a more specific p53‐MDM2 inhibitor RG7112 were used.^[^ ^36^, ^37^ ^]^ MG132 treatment increased p53/p21 protein levels, and protected *SETD8^C302R^* mutant cells from MK1775 (Figure 5B,C). Likewise, RG7112 prevented p53 degradation more effectively, and nearly abrogated the MK1775 sensitization effect of *SETD8^C302R^* (Figure 5D,E). To this end, we conclude that SETD8 is essential in maintaining genomic stability, and that the patient‐derived *SETD8^C302R^* mutation leads to p21 deficiency and thus a defect in safeguarding genome integrity, which confers increased sensitivity to WEE1 inhibition. This revelation provides a potential therapeutic approach for future cancer patients carrying the same mutations or functional defects (Figure 5F).

## 3 Discussion

In this study, by comparing an IMT patient to her healthy twin sister, we screen out a SNV of *SETD8^C302R^*, which is first reported to be related to IMT. In functional studies, we demonstrate that SETD8 plays an essential role in promoting faithful DNA replication, loss of which leads to replication fork perturbation and replication‐dependent DNA damage, which is in line with previous findings that co‐depletion of SETD8 and replication‐associated proteins such as Cdc45 and MCM4 can reduce DNA damage.^[^ ^38^ ^]^ The patient‐derived *SETD8^C302R^* mutation does not show any evident impact on genomic instability under unstressed conditions, but downregulates p53/p21 pathway and thus leads to increased sensitivity to WEE1 inhibition. Our results underscore the importance of identifying rare mutations and revealing their functional consequences, which hold the key to improve the therapeutic outcomes of the affected patients. This is the first study to provide a practical treatment method through genetic analysis followed by functional studies, which has not been achieved in other studies. Of note, our work mainly employed U2OS cells to investigate the effect of *SETD8^C302R^*, which deserves further validation using lung inflammatory myofibroblastoma cells when they are available. Moreover, future preclinical and clinical studies may continue to investigate the translational potential of WEE1 inhibitor for cancer patients carrying *SETD8^C302R^*, e.g., by using genetically modified animal models or conducting retrospective analysis on results from clinical trials of WEE1 inhibitors.

Because the salient enzymatic function of SETD8 is monomethylation of H4, we initially expected that the patient‐derived *SETD8^C302R^* mutation might affect the H4K20me1 formation. However, despite locating in the catalytic domain of SETD8, *SETD8^C302R^* does not alter the overall level of H4K20me1. Instead, p53, a non‐histone substrate of SETD8,^[^ ^31^ ^]^ seems to be more affected by this mutation, as observed that p21, the downstream effector of p53, was prominently downregulated in *SETD8^C302R^* cells (Figure 3G). Because p21 expression is significantly downregulated at the mRNA level, we posit that *SETD8^C302R^* may impinge on p21 gene transcription activity. Indeed, *SETD8^C302R^* evidently reduced p53 binding to the promoter of p21 gene (Figure 3I), which might contribute to the reduced *p21* mRNA level. Whether this effect of *SETD8^C302R^* is caused by altered p53 methylation level remains to be investigated. Besides, p53‐independent regulatory pathways for p21, such as the HRAS–Raf–Mapk pathway, and other post‐transcriptional mechanisms affecting *p21* mRNA stability,^[^ ^39^, ^40^ ^]^ may also need further studies.

The increased sensitivity of *SETD8^C302R^* mutant cells to WEE1 inhibition may be attributed to both replication perturbation and checkpoint dysfunction. p21 is reported to be vital for promoting nascent DNA elongation, and WEE1 is required for suppressing aberrant CDK2 activation and ensuring replication fork speed;^[^ ^41^ ^]^ therefore, p21 deficiency and WEE1 inhibition may synergistically cause severe replication defects in *SETD8^C302R^* mutant cells (Figure 4E,F), leading to irreparable genetic lesions. However, *SETD8^C302R^* mutant cells are less sensitive to cisplatin and camptothecin than to MK1775, which means there are other mechanisms for increased sensitivity of *SETD8^C302R^* to WEE1 inhibition. p21 is a pivotal downstream effector of p53, mainly mediating G1‐phase arrest in response to various stimuli. Insufficient p21 in tumor cells results in a defective G1 checkpoint, which impairs the ability of cells to halt the cell cycle to repair DNA damage before replication. Because WEE1 is a tyrosine kinase that is essential for the G2‐M cell cycle checkpoint, inhibition of WEE1 will inactivate the G2 checkpoint and permits cell division that may pass DNA damage to the next cell cycle, which is especially deleterious in *SETD8^C302R^* cells since they lack an efficient G1 checkpoint to prevent the further accumulation of damage. The present study was consented by the patient's sisters and their families, and approved by the Ethics Committee of the Emergency General Hospital of Beijing.

## 4 Conclusion

We have demonstrated the utility of WGS on MZ twins for identifying rare pathogenic mutations, which can be followed by functional studies to unravel pathogenic mechanisms and to develop effective therapeutics. Our data show that *SETD8^C302R^* cancer cells are more sensitive to WEE1 inhibition compared with *SETD8^WT^* cells, which present a promising therapeutic approach for cancer patients carrying this mutation.

## 5 Experimental Section

Detailed methods and statistical analysis are provided in the Supporting Information. The patient study was consented by the sisters and their families, and approved by the Ethics Committee of the Emergency General Hospital of Beijing (Project Number: K19‐23).

## Conflict of Interest

The authors declare no conflict of interest.

## Supporting information

Supporting Information
