# Supplementary material for: SETD8C302R Mutation Revealed from Myofibroblastoma‐Discordant Monozygotic Twins Leads to p53/p21 Deficit and WEE1 Inhibitor Sensitivity
Source: Adv Sci (Weinh). 2020 Aug 5;7(19):2001041. doi: 10.1002/advs.202001041 (PMC7539211; doi:10.1002/advs.202001041)
Supplement: Supplementary file 1 — Supporting Information [file ADVS-7-2001041-s001.pdf]

© 2020 Wiley-VCH GmbH

ADVANCED SCIENCE
Open Access

Supporting Information

for *Adv. Sci.,* DOI: 10.1002/advs.202001041

SETD8^{C302R} **Mutation Revealed from Myofibroblastoma-Discordant Monozygotic Twins Leads to p53/p21 Deficit and WEE1 Inhibitor Sensitivity**

Miao Li, Hongwu Wang, Hongwei Liao, Jiaxin Shen, Yinfang Wu, Yanping Wu, Qingyu Weng, Chen Zhu, Xinwei Geng, Fen Lan, Yang Xia, Bin Zhang, Hang Zou, Nan Zhang, Yunzhi Zhou, Zhihua Chen, Huahao Shen, Songmin Ying,* and Wen Li**

Supporting Information

Title

SETD8^{C302R} Mutation Revealed from Myofibroblastoma-Discordant Monozygotic Twins Leads to p53/p21 Deficit and WEE1 Inhibitor Sensitivity

Miao Li#, Hongwu Wang#, Hongwei Liao#, Jiaxin Shen, Yinfang Wu, Yanping Wu, Qingyu Weng, Chen Zhu, Xinwei Geng, Fen Lan, Yang Xia, Bin Zhang, Heng Zou, Nan Zhang, Yunzhi Zhou, Zhihua Chen, Huahao Shen, Songmin Ying*, Wen Li**

Experimental Section

Sample description and whole genome sequencing of the monozygotic twin pair

A 10-year-old girl was admitted to the hospital for her cough and severe dyspnea. Chest computed tomography (CT) scan revealed a neoplasm that blocked the right main bronchus and most of the left main bronchus. Soon she was pathologically diagnosed with pulmonary right bronchial inflammatory fibroblastoma while her monozygotic twin sister remained healthy. To explore the potential tumor-related genomic alterations derive from the patient, we obtained whole-genome sequencing data covering 99.02% and 99.07% of the two genomes using peripheral blood cells from the twin pair by Illumina Hiseq at Novogene (Beijing, China), which was consented by the sisters and their families, and approved by the Ethics Committee of the Emergency General Hospital of Beijing (Project Number: K19-23). Valid sequencing data are aligned to the reference genome via BWA[1] and Samblaster. With

the genome data of her healthy twin sister as a germline and normal tissue control, patient specific genetic alterations including single nucleotide variants (SNVs), insert and deletion (InDels), copy number alterations (CNAs) and structural variants (SVs) were reserved. After annotation and interpretation by ANOVAR, missense mutations in exonic and splicing regions of the patient were screened out and scored using Mutation Taster. At last, we picked up four potentially deleterious SNVs in *FRG1*, *SETD8*, *BEGAIN*, *KIR2DL3* with general mutation frequency lower than one in 70,000 for additional cellular-based experiments.

Cell culture

All cell lines were purchased from ATCC and cultured in DMEM (Hyclone, U2OS, A549) or RPMI 1640 (Gibco, HBE) supplemented with 10% fetal bovine serum (Gibco) at 37°Cunder an atmosphere containing 5% carbon dioxide.

Antibodies and agents

Primary antibodies used for immunofluorescence and western blot as follows: mouse anti-γH2AX (05-636, Millipore, 1:1000), mouse anti-RPA23 (ab2175, Abcam, 1:1000), rabbit anti-phospho-RPA (ab109394, Abcam, 1:50000), rabbit anti-SETD8 (06-1304, Millipore, 1:500), mouse anti-p53 (sc-126, Santa Cruz, 1:500), rabbit anti-p21 (10355-I-AP, Proteintech, 1:1000), mouse anti-IgG (sc-69786, Santa Cruz) and mouse anti-β-Actin (E021020-01, Earthox, 1:1000). Agents as follows: MK1775 (Selleck), camptothecine (Selleck), cisplatin (Selleck), CHK1 inhibitor (Selleck), RG7112 (MCE), MG132 (MCE), puromycin (Beyotime).

siRNAs transfection

Cells were seeded in 6-well plates at a density of 5×10^5 per well. Transfection reagent for siRNA was from SignaGen (SL100568). siRNAs targeting *FRG1*, *BEGAIN*, *KIR2DL3* were from Santa Cruz. *SETD8* siRNAs sequences as follows: 5'-GCAACUAGAGAGACAAAUCUU-3' and 5'-CACACUUAAUCUUAGCGGATT-3'. All transfections were handled following the manufacturer's protocols.

Real-Time PCR analysis

Total RNA was extracted using Trizol (Takara) and quantitative reverse transcription-PCR (qRT-PCR) was performed. Primer sequences as follows: *SETD8*-forward 5'-GGGAAACCATTAGCCGG

AATC-3, *SETD8*-reverse 5'-CTTCATGGCGCTCCGTACTG-3'; *p21*-forward 5'-TGTCCGTCAGAACCCA

TGC-3', *p21*-reverse 5'-AAAGTCGAAGTTCCATCGCTC-3'; *p53*-forward 5'-CACTGCCATGGAGGAGC

CGCA-3', *p53*-reverse 5'-GTCACAGACTTGGCTGTCCCAG-3'; *FRG*1-forward 5'-TTGTTGTTGGGCGT

TCAGATG-3', *FRG1*-reverse 5'-GCTTCTATGTCCCCTGCTTCATT-3'; *BEGAIN*-forward 5'-CGACAAGCTGTCAGAGGACAA-3', *BEGAIN*-reverse 5'-GCGCTCCTGGAAATCCGAG-3'; *KIR2DL3*-forward 5'-CCTTCATCGCTGGTGCTG-3', *KIR2DL3*-reverse 5'-CAGGAGACAACTTTGGATCA-3'; *b-Actin*-forward 5'-CATGTACGTTGCTATCCAGGC-3', *b-Actin*-reverse 5'-CTCCTTAATGTCACGCACG

AT-3'. All data were normalized to the housekeeping gene *β-Actin*.

Flow cytometry

Cells were seeded in 6-well plates at a density of 2×10^5 cells per well. After siRNA transfection for 48h or addition of drugs for 48h, cells were collected by centrifugation at 1000rpm for 5min at 4°C, followed by three times wash with PBS. Annexin V (70-APCC101-100, MultiSciences) dye was used to label the samples by incubating for 15min at room temperature (*RT*). Fluorescent signal was analyzed on a Beckman flow cytometer using CytExpert software.

Immunofluorescence

Cells were seeded in 24-well plates and treated as previously indicated. Indirect immunofluorescence was used to study the expression of γH2AX and RPA. Cells were fixed in 4% formaldehyde/PBS at *RT* for 15min, permeabilized with 0.5% Triton X-100/PBS for 20min and blocked by incubation with 3% BSA in PBS for at least 1h at *RT*. To proceed, the samples were incubated with indicated primary antibodies at 4°C overnight, followed by incubation with secondary antibodies conjugated with Alexa Fluor 488 or 555 (Life Technologies) at *RT* for 30min. Nuclei were stained with DAPI. For quantification of γH2AX foci in replicating and non-replicating cells, 10μM EdU was pulse-labeled for 30min before harvesting to identify replicating cells. After the primary antibody for γH2AX was applied, cells were stained for EdU using the Click-iT-EdU kit (C10337, Invitrogen), according to the manufacturer's protocol, standard immunostaining using the secondary antibody was performed. Images were

visualized using an automated Nikon Eclipse Ni microscope with Nikons-Elements software (Nikon Instruments). At least 300 cells from random views were imaged and analyzed for each sample.

Western blot analysis

Cells were lysed in ice cold RIPA lysis buffer (Beyotime) supplemented with proteinase inhibitor Cocktail (Roche) and phospho-stop cocktail (Roche). Cell lysates were sonicated at 80% power every 8s for three times and then clarified by centrifugation at 17000rpm for 10min. Total protein content was measured using the BCA assay (ThermoFisher) before 5×loading buffer (bromophenol blue (0.25%), glycerol (50%), SDS (sodium dodecyl sulfate; 10%), Tris-Cl (0.25M, pH 6.8), mercaptoethanol (3.6%)) was added and protein samples were boiled at 100°C for 8min. Total protein extracts were loaded and separated on 10–15% SDS-PAGE gels. Proteins were then blotted onto PVDF membranes (Millipore) using transfer buffer (25mM Tris base, 189mM glycine, 20% methanol) at 4°C. Membranes were blocked with 5% non-fat milk in TBST for 1h at *RT*, followed by incubation with primary antibody at 4°C overnight. The secondary antibodies (Earthox) were incubated for 1h at *RT*. Immunoreactive bands were visualized using an Odessey scanner.

DNA fiber assays

DNA fiber spreading was performed as described previously.[2] Briefly, subconfluent cells were sequentially labeled with 25μM CldU (Sigma) and 250μM IdU (Sigma) for 20min. Cells were then collected and suspended in cold PBS at a density of 5×10^5/mL. To spread the DNA fibers,

2μL cell suspension was loaded on the slide and incubated for 5min and gently mixed with 7μL lysis buffer (200mM Tris-HCl, pH 7.5, 50mM EDTA, 0.5% SDS) before incubation for another 2min. Slides were then tilted at 15° angle to run the lysis buffer down the bottom slowly, and were then air dried, fixed in methanol and acetic acid (3:1) for 10min and again allowed to dry. The slides were rehydrated in PBS and denatured in 2.5M HCl for 80min at *RT*. Glass slides were then blocked with 1% BSA containing 0.1% Tween-20 for 1h at *RT*, followed by immunostaining with mouse anti-BrdU to detect IdU (#347580, Becton Dickinson, 1:200), rat anti-BrdU (ab6326, Abcam, 1:1000) to detect CldU, goat anti-mouse secondary antibody conjugated to Alexa Fluor 488 (Life technologies) and goat anti-rat secondary antibody conjugated to Alexa Fluor 594 (Abcam). Nascent DNA fibers were visualized on the Nikon Eclipse Ni microscope with Nikon Elements software (Nikon Instruments) using a 60× oil-immersion objective. Fiber length measurement and replication structure fractionations were performed using Nikon Elements software. The replication speed was calculated according to the labeling time. For each sample, at least 200 replication structures were counted.

Comet assays

Cells were cultured in 6-well plates and treated as indicated. After digestion by 0.25% trypsin containing EDTA, cells were collected by centrifugation at 1000rpm for 5min and resuspended in ice-cold PBS at a density of 1×10^6/mL. Adhesive slides were pre-warmed at 37°Cfor 30min. The base layer was tiled with 100μL 0.5% dissolved normal melting point agarose (NMA) on pre-warmed slides and solidified at 4°Cfor 10min. 10μL cell suspension was immobilized in a

bed of 75μL0.7% low melting point agarose (LMA) on the base layer, followed by solidification at 4°Cfor 10min. The top layer was tiled with 100μL 0.7% LMA and solidified at 4°Cfor 30min. Next, slides were immersed in lysis buffer (KeyGEN) at 4°C for 1.5h and treated with alkali (1mM EDTA, 300mM NaOH) to unwind and denature the DNA before electrophoresis, which lasted for 25min at voltage 25V. After electrophoresis slides were neutralized with 0.4mM Tris-HCl (pH 7.5) three times at 4°C followed by PI staining. Images were obtained on the Nikon Eclipse Ni microscope as mentioned above. Olive tail moments were analyzed by Casplab. For each sample, at least 50 cells were measured.

Generation of CRISPR *SETD8* mutation cells

Two single-guide RNAs (sgRNAs) targeted near the T>C single nucleotide variant were cloned into the sgRNA/Cas9 expression vector pEP330X. sgRNA sequences as follows: 5'-GCAACTAGAGAGA CAAATCGCCT-3' and 5'-GCTGTGATTGATCAGTCTTCCT-3'. The donor vector was constructed with the T>C single nucleotide variant containing sequence flanked by 150 and 350 base pair homology arms. To prevent the donor from being cut by Cas9, sequences recognized by sgRNAs were synonymously mutated as follows: 5'-GCAACGAGGGAGACCAATCG
TCT-3' and 5'-GGGAAGGCTTATAAACCATAGC-3'. Donor and sgRNAs were transiently co-transfected to U2OS cells for 36h followed by 1μg mL^{-1} puromycin selection for 48h. Point mutation clones were expanded from single cells and validated by PCR and DNA sequencing.

Chromatin Immunoprecipitation Assays

ChIP assays were performed using the SimpleChIP enzymatic ChIP kit (9003, Cell Signaling Technology) according to the manufacturer's instructions. In brief, cells were cross-linked with 1% formaldehyde for 10min at *RT* and nuclei were purified. For chromatin digestion, micrococcal nuclease was added for 20min at 37°C followed by sonication (three times, 10s each). The DNA–protein complexes were immunoprecipitated overnight at 4°C using 2 μg of p53 antibody (DO-1, Santa Cruz) or normal mouse IgG (Santa Cruz) as the negative control. Immunoprecipitated chromatin was incubated with protein G beads, washed and eluted at 65°C After reversal of formaldehyde crosslinks and purification of DNA, the bound DNA fragments were analyzed by quantitative real-time PCR using SimpleChIP universal qPCR master mix (88989, Cell Signaling Technology). Primers of p21 promoter: 5′-GTGGCTCTGATTGGCTTTCTG-3′ and 5′-CTGAAAACAGGCAGCCCAAG-3′.

Statistical Analysis

Continuous variables were presented as mean ± SEM. For each statistical analysis, at least 3 independent samples were contained. Statistical significance between two-group was calculated using two-tailed student's t test. Statistical evaluation of more than two groups was performed using one–way ANOVA followed by Dunnett's multiple comparison post-tests. For grouped analysis, two-way ANOVA followed by Sidak's or Tukey's multiple comparison post-tests was used. P values<0.05 were considered significant. Statistical analysis was carried out using Microsoft Excel and Graphpad Prism 8.0.

References

[1] H. Li, R. Durbin, *Bioinformatics* **2009**, 25, 1754.

[2] D.A. Jackson, A. Pombo, *J. Cell Biol.* **1998**, 140, 1285.

Supplementary Figures

Figure S1 SETD8 knockdown retards cell proliferation and induces apoptosis.

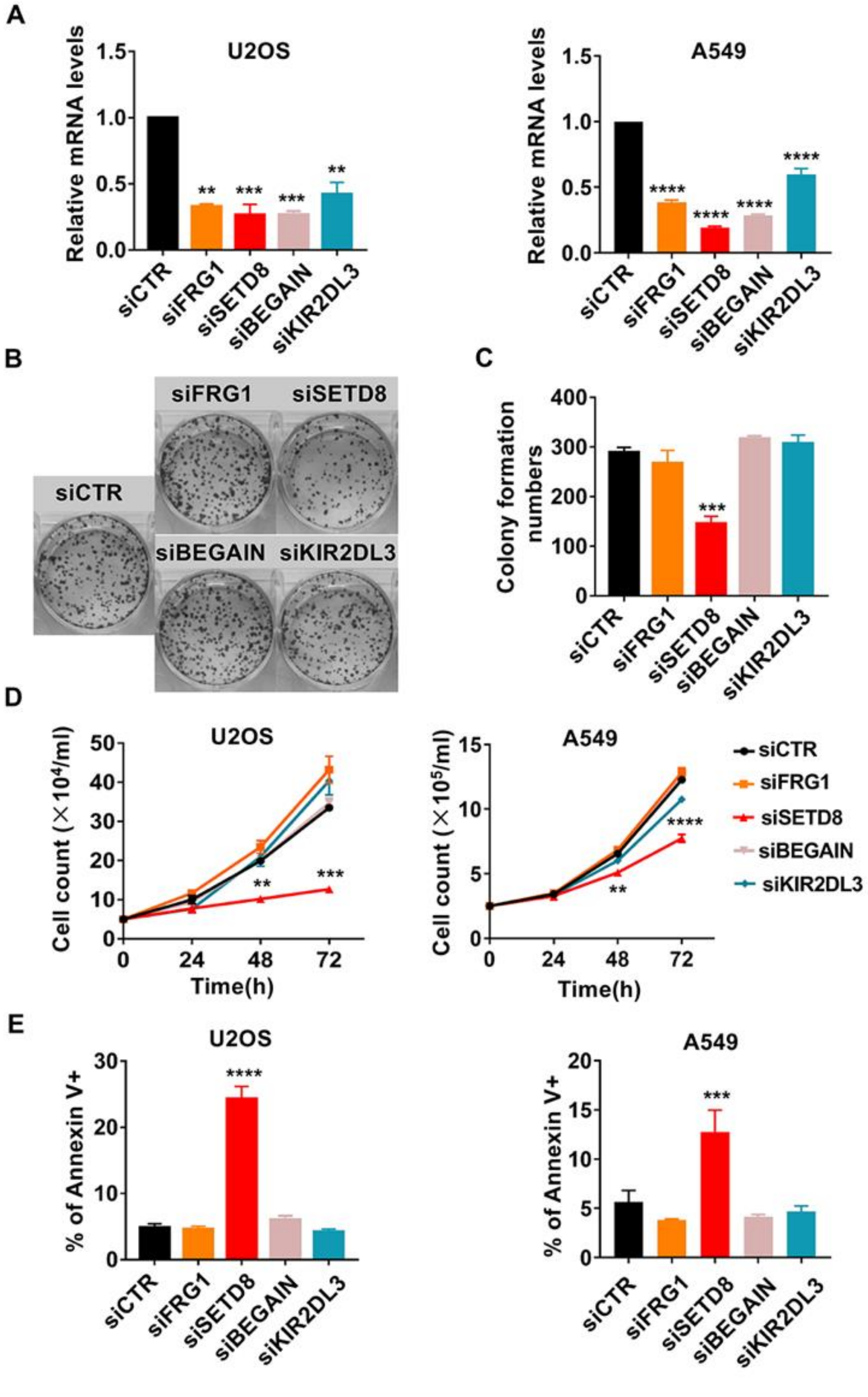

A Knockdown efficiency quantified using mRNA levels relative to control after specific siRNAs targeting *FRG1*, *SETD8*, *BEGAIN*, and *KIR2DL3* were transfected to U2OS and A549 cells for 48h respectively (at least 4 independent samples). **B** Representative images of clone formation

assays in U2OS cells transfected with siRNAs. **C** Quantification of the data from **B** (n=3 independent samples). **D** Cell counting for U2OS and A549 cells transfected with siRNAs for 24h, 48h and 72h (at least 3 independent samples). **E** Apoptosis analysis of U2OS and A549 cells transfected with siRNAs for 72h by flow cytometry (at least 3 independent samples). Data are presented as means ± SEM. One-way ANOVA followed by Dunnett's multiple comparison post-tests was performed in **A**, **C**, **D**, **E**. **: p<0.01; ***: p<0.001; ****: p<0.0001.

Figure S2. SETD8 deficiency in HBE cells also leads to spontaneous DNA damage

response.

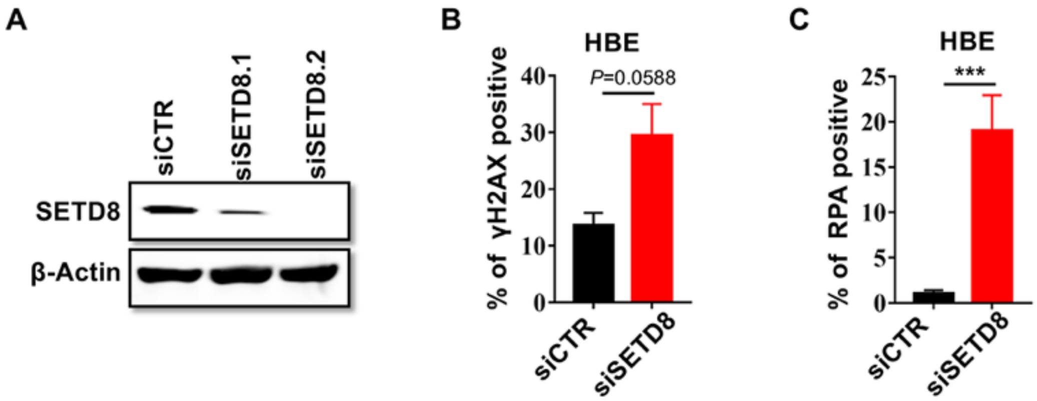

A Immunoblot analysis of two different SETD8 siRNAs knockdown efficiencies in U2OS. β-Actin was used as a loading control. **B-C** Quantification of γH2AX and RPA in HBE cells treated with siSETD8.2 for 48h. Cells with over 5 γH2AX or RPA foci were counted as positive cells (at least 3 independent samples). Data are presented as means ± SEM. Two-tailed student's *t*-tests were performed in **B**, **C**. ***: $p<0.001$.

Figure S3. ***SETD8^{C302R}*** **mutation do not affect cell proliferation and apoptosis.**

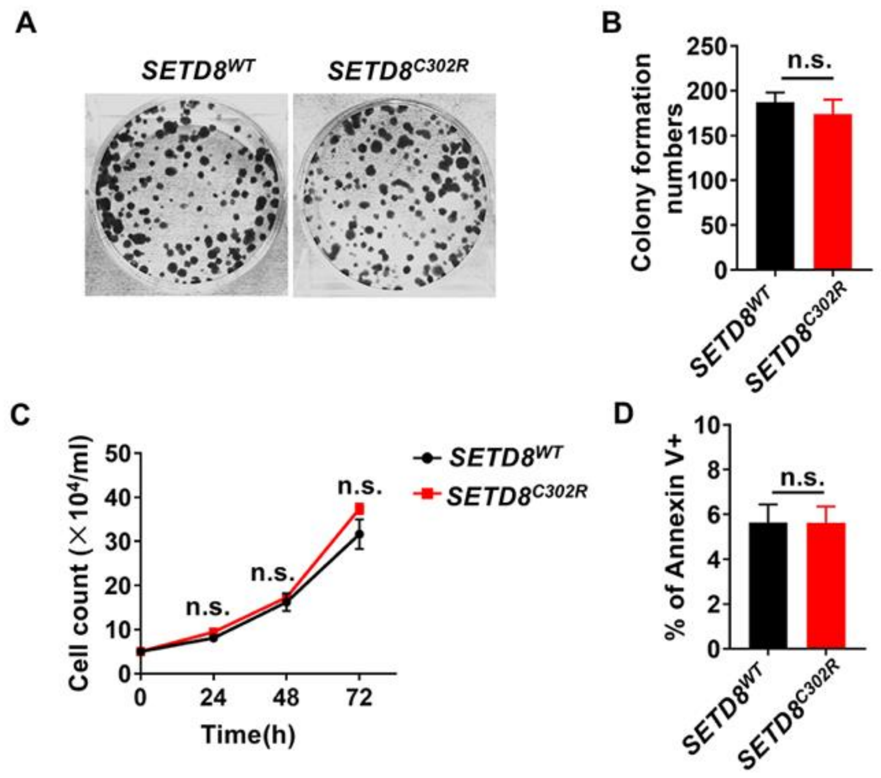

A Representative images of clone formation assays for *SETD8^{WT}* and *SETD8^{C302R}* mutant cells. **B** Quantification of the data from A (n=3 independent samples). **C** Cell counting for *SETD8^{WT}* and *SETD8^{C302R}* mutant cells at 24h, 48h and 72h after seeding at a density of 5×10^5 per well (n=6 independent samples). **D** Apoptosis analysis of *SETD8^{WT}* and *SETD8^{C302R}* mutant cells by flow cytometry (n=6 independent samples). Data are presented as means ± SEM. Two-tailed student's *t*-tests were performed in **B-D**. n.s.: not significant.

Figure S4. *SETD8^{C302R}* mutant cells are not sensitive obviously to camptothecin and cisplatin.

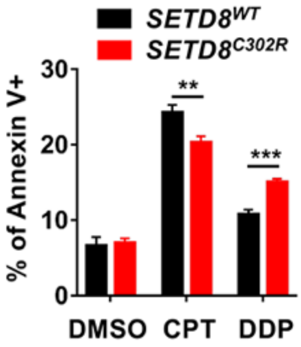

Apoptosis analysis of cells treated with 2μM camptothecin or 2μg mL^{-1} cisplatin for 48h. Apoptotic cells were labeled with Annexin V (at least 4 independent samples). Data are presented as means ± SEM. Two-way ANOVA followed by Sidak's multiple comparison post-tests were performed. **: $p<0.01$, ***: $p<0.001$.
